# Patellar sleeve avulsion fracture in a patient with Sinding-Larsen-Johansson syndrome: a case report

**DOI:** 10.1186/s12891-020-03297-z

**Published:** 2020-04-23

**Authors:** Andrés Schmidt-Hebbel, Felipe Eggers, Vincent Schütte, Andrea Achtnich, Andreas B. Imhoff

**Affiliations:** 1 Clínica Alemana de Santiago, Departamento de Ortopedia y Traumatología, Av. Vitacura 5951, Santiago de, Chile; 2 Technical University of Munich. Klinikum rechts der Isar, Abt. f. Sportorthopädie- Ismaninger Str. 22, 81675 Munich, Germany; 3 Klinik für Unfall- und Wiederherstellungschirurgie, BG Klinikum Bergmannstrost, Marseburger Str. 165, 06112 Halle/Saale, Germany

**Keywords:** Sleeve fracture, Patellar avulsion, Sinding-Larson-Johansson, Fiber tape, Surgical technique

## Abstract

**Background:**

Patellar sleeve avulsion (PSA) fractures are rare injuries that occur in in skeletally immature patients. Initial diagnosis is key to a successful outcome, as these injuries are easily overlooked on plain radiographs with poor results well documented from delayed management. High index of suspicion from the mechanism of injury, thorough clinical examination and Magnetic Resonance Imaging (MRI) help to avoid misdiagnosis.

**Case presentation:**

The case of a 12-year-old male athlete with an acute PSA after a conservative treatment of a SLJ syndrome is described. The patient was referred to our clinic due to severe pain and loss of function after performing a high jump. Plain radiographs (X-ray) and MRI confirmed an inferior pole PSA which was fixed with double trans osseous ultra-high strength tapes. At the 3-month follow- up visit the patient was able to ambulate brace free. At 2-years follow up the patient was able to play soccer and ice hockey. To our knowledge, there are no case reports of inferior pole PSA with prior SLJ syndrome described in literature.

**Conclusions:**

Early clinical suspicion and distinguishing this PSA from other enchondral ossification disorders around the knee is critical to avoid misdiagnosis. Whether SLJ syndrome increases the risk of sustaining a PSA is still not clear. Trans osseous fixation with suture tapes leads to good functional results in a young athlete with inferior pole PSA.

## Introduction

Patellar sleeve avulsions (PSA) are rare injuries that occur in children and adolescents, and represent less than 1% of all pediatric fractures [1, 2]. Most reported cases of PSA involve the inferior patellar pole, but less frequent bilateral and superior pole fractures have also been reported [3, 4]. The peak incidence of sleeve fractures in children is 12.7 years, with a range of 8 to 16 years [5–7]. At this age the patella is more hypermobile, and the relatively high cartilage-bone ratio at the transformation zone makes it more vulnerable to acute and chronic eccentric loads and shear forces. The main mechanism of injury in PSA is indirect, acute forceful muscle contraction of the quadriceps muscle typically seen during jumping activities [8, 9].

In the literature, there is a limited data regarding treatment options and outcome for PSA. The main goal is to achieve a functional extensor mechanism, which can be accomplished through conservative treatment in the cases of non-displaced avulsion. Displaced PSA treated non operatively may lead to complications like patella alta, anterior knee pain or loss of quadriceps function, and therefore it is important to be aware of this rare injury. Subtle bony avulsions on plain radiographs might be easily overlooked. A high index of suspicion from the mechanism of injury, thorough clinical examination and Magnetic Resonance Imaging (MRI) help to avoid misdiagnosis [2].

The present case report describes relevant clinical details, imaging and the surgical treatment of an inferior pole PSA fracture in a 12-year-old athlete with previous Sinding-Larsen-Johansson (SLJ) syndrome. To our knowledge, there are no case reports of inferior pole PSA with prior SLJ syndrome published in literature. The patient and his family were informed that data concerning the case would be submitted for publication, and our institutional review board approved the study.

## Case presentation

**Fig. 1:**
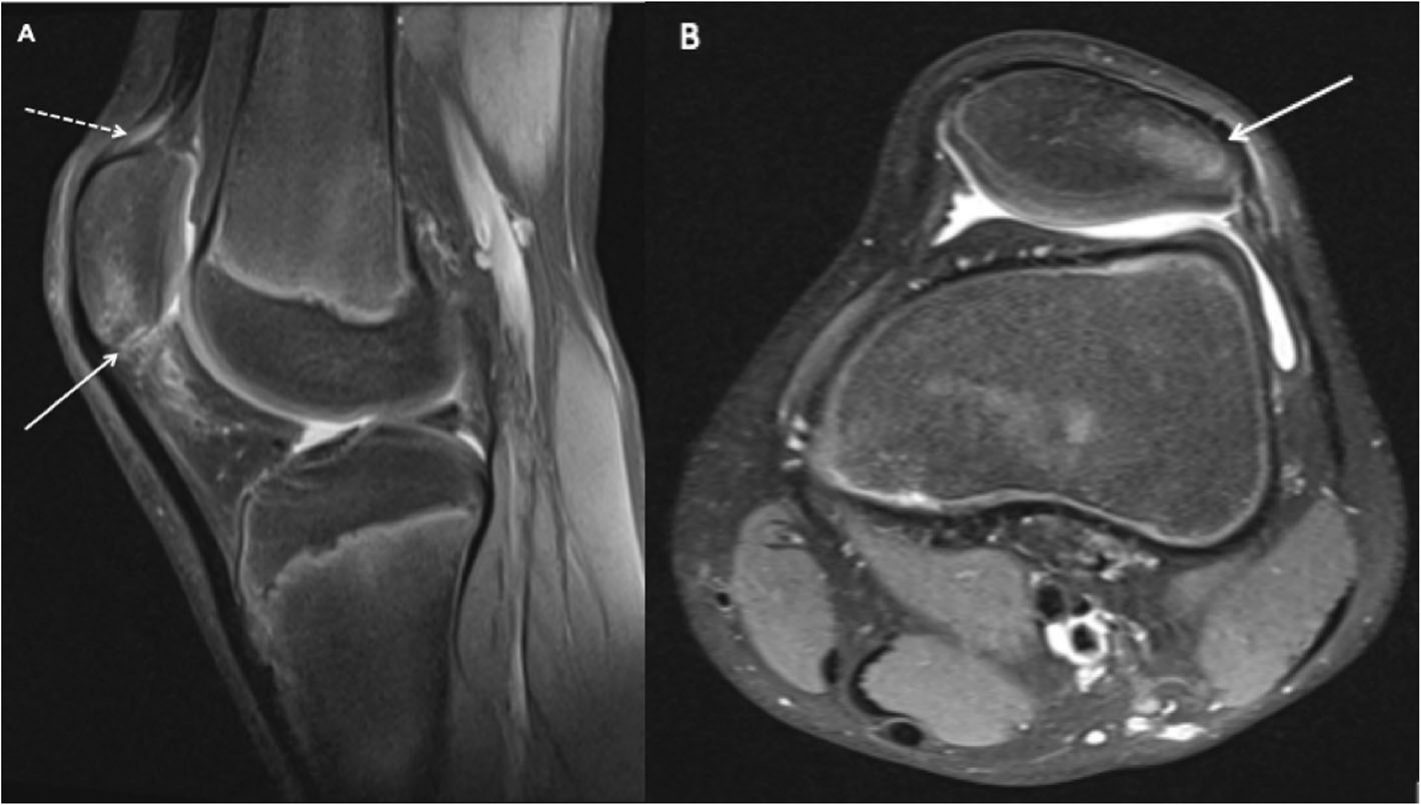
T2-weighted MRI 4 months prior to acute injury. **a**: Increased bone signal of inferior patellar pole (straight white line) and quadriceps tendinosis (dotted white line). **b**: Increased bone signal in lateral patellar facet compatible with bone edema (straight white line)

We describe the case of a healthy 12-year-old male athlete with SLJ syndrome and acute PSA. The patient reported chronic anterior knee pain 4 months before related to repetitive high jumping activity. Clinical findings and MRI were compatible with SLJ syndrome (Fig. 1). The patient’s activity level was reduced to a minimum, and after 3 months of conservative treatment he initiated high jumping activity.

**Fig. 2:**
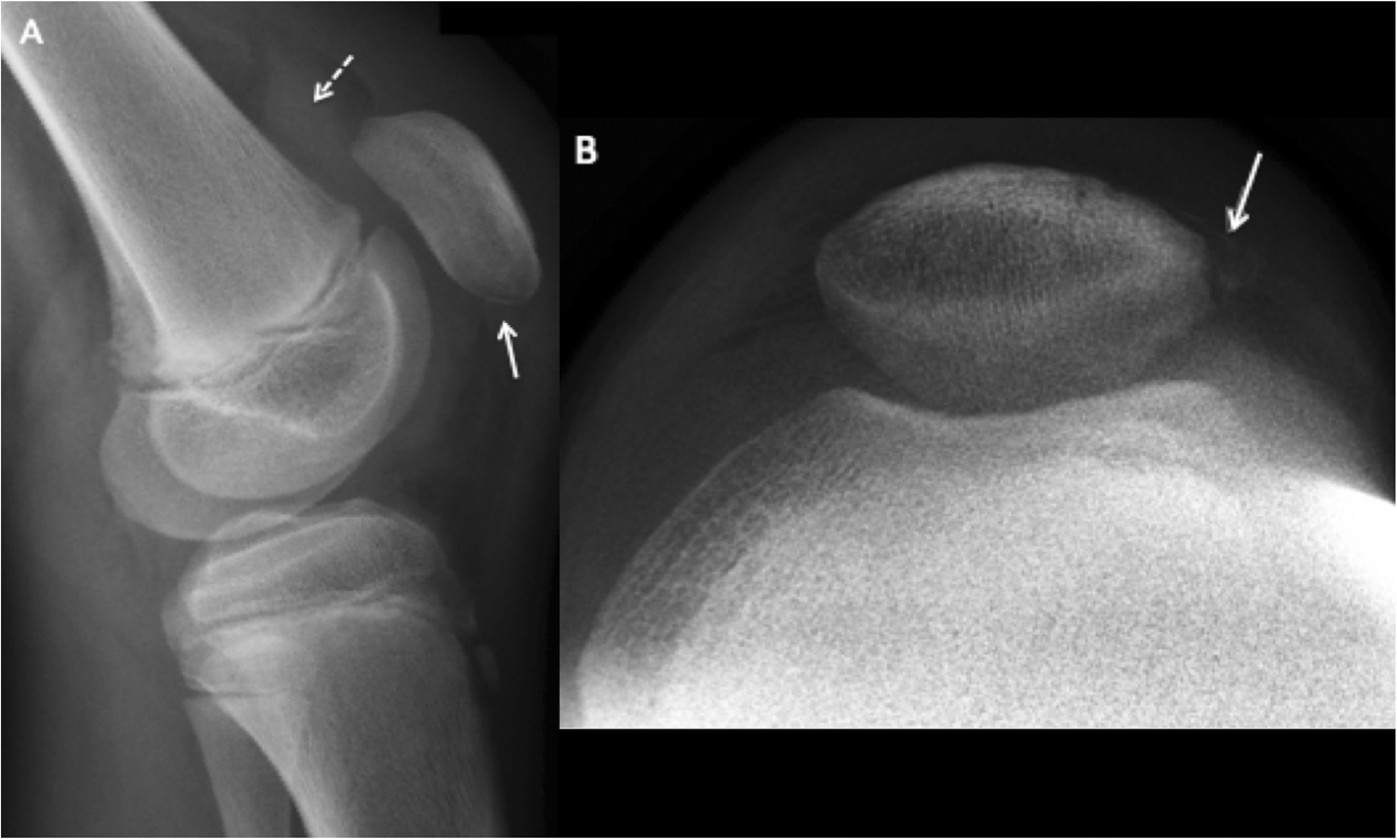
**a**: Minimal avulsion fracture of inferior patellar pole (straight white line) and large intra-articular effusion (dotted white line) on lateral X-ray. **b**: Sunrise view demonstrating lateral retinacular avulsion (straight white line)

**Fig. 3:**
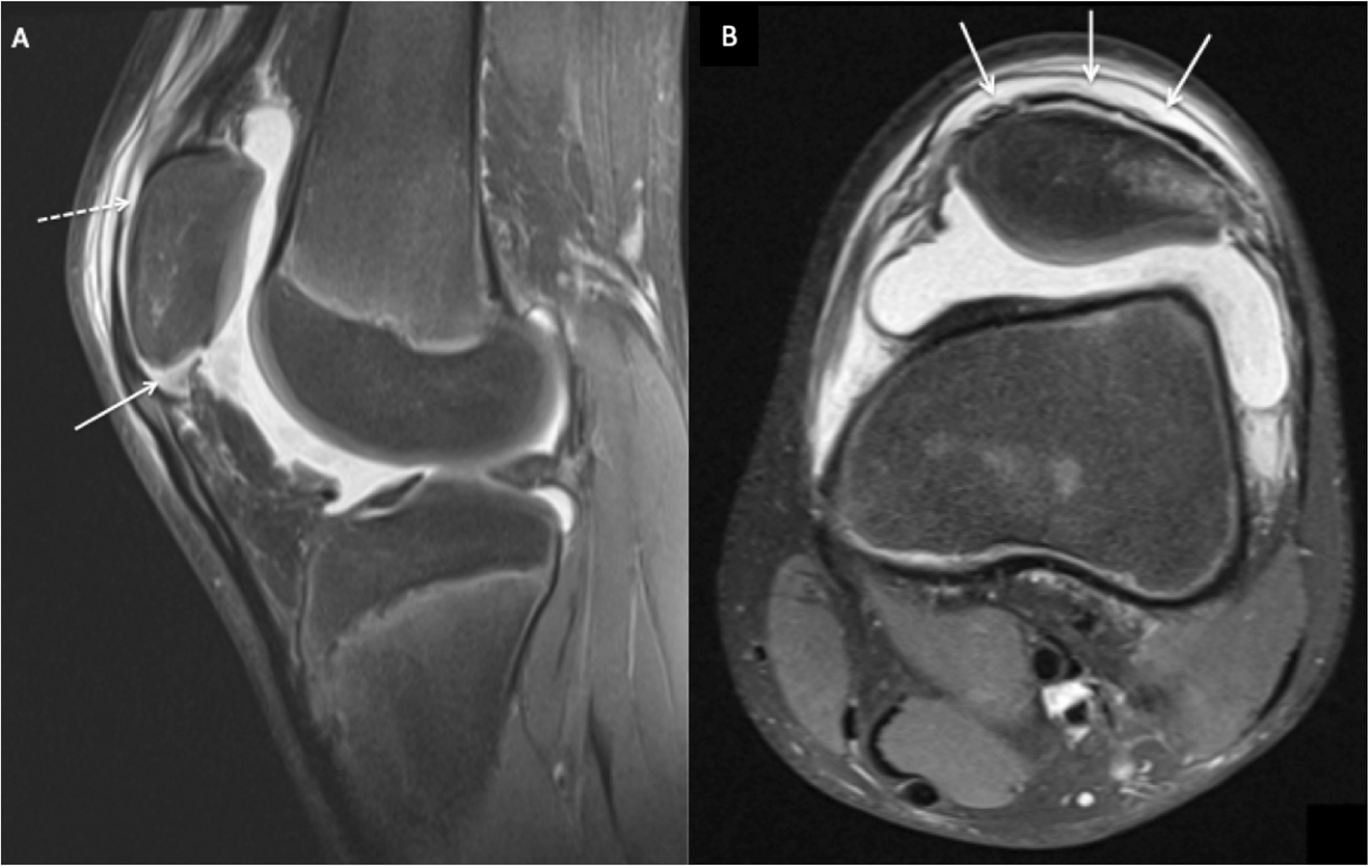
**a**: Sagittal T2-weighted MRI after acute injury showing patellar tendon sleeve avulsion (straight white line) with sub periosteal-fluid surrounding the patella (dotted white line). **b**: Axial T2- weighted MRI demonstrating sub-periosteal fluid (straight white line) communicating with the intraarticular effusion

Four months following the first symptoms of SLJ syndrome, the patient reported severe knee pain and loss of function after performing a high jump. He was referred to our outpatient clinic with a swollen, painful left knee and difficulty with weight bearing. Examination revealed haemarthrosis with tenderness over the patella, limited range of motion and a palpable defect of the patellar tendon. The patient was unable to perform a straight-leg raise. Plain radiographs (X-ray) and MRI confirmed an inferior pole PSA (Figs. 2 and 3), and surgery was indicated.

**Fig. 4:**
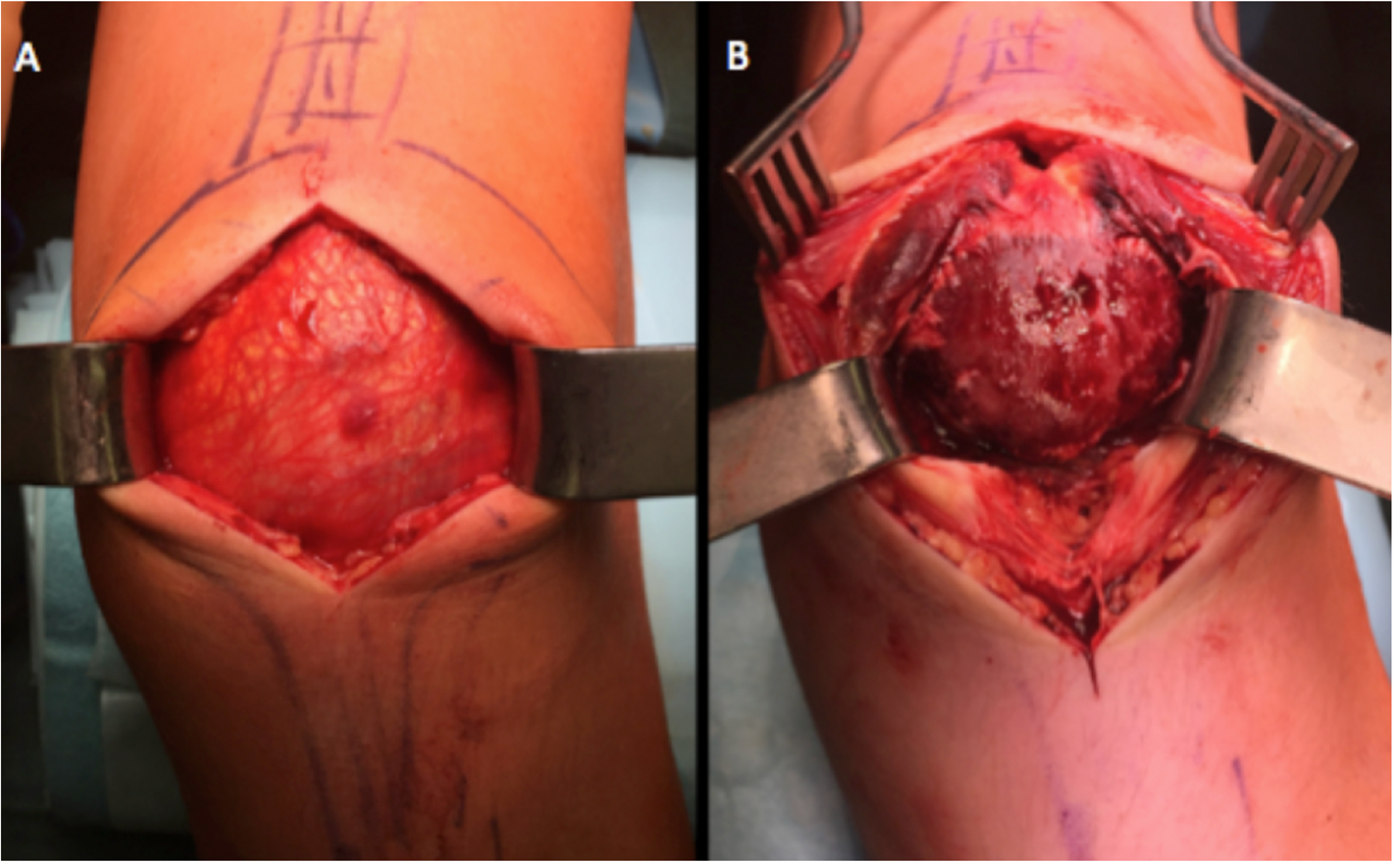
Intraoperative pictures **a**: Intact prepatellar bursa. **b**: Prepatellar bursa and periosteum sharply dissected. The patellar tendon was avulsed off the distal pole and anterior aspect of blood infiltrated patella, with an intact proximal patellar pole and quadriceps tendon

**Fig. 5:**
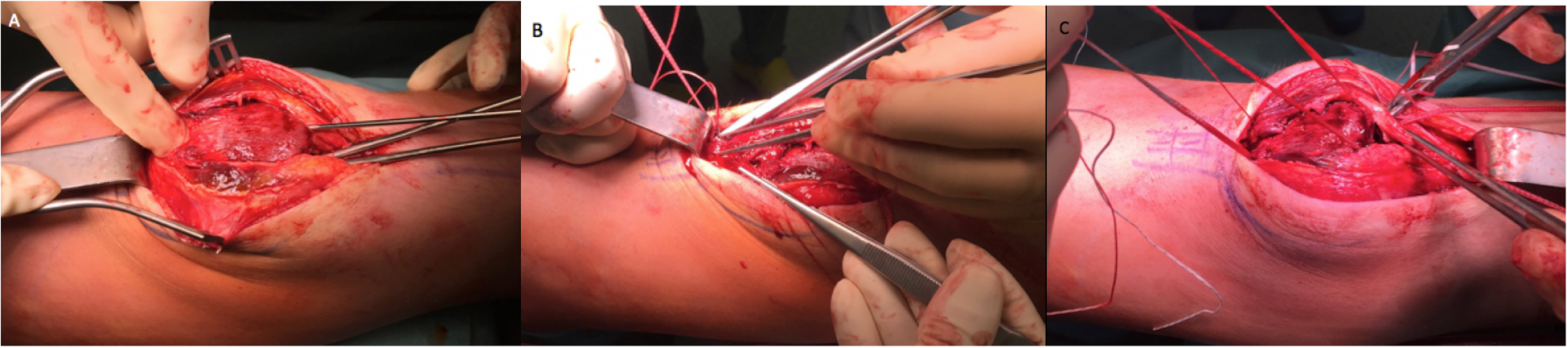
**a**: Two vertical patella bone tunnels were drilled from distal to proximal. **b**: One end of a FiberTape® was passed through the lateral tunnel from distal to proximal and whipstitched the distal quadriceps tendon. **c**: A TigerTape™ suture (Arthrex, Naples, Florida) was passed from distal to proximal, one end in each tunnel, and both ends were held over the proximal pole. The FiberTape® was shuttled down the medial patellar bone tunnel and whipstitched the proximal patellar tendon where it was tied down in 90° of flexion

**Fig. 6:**
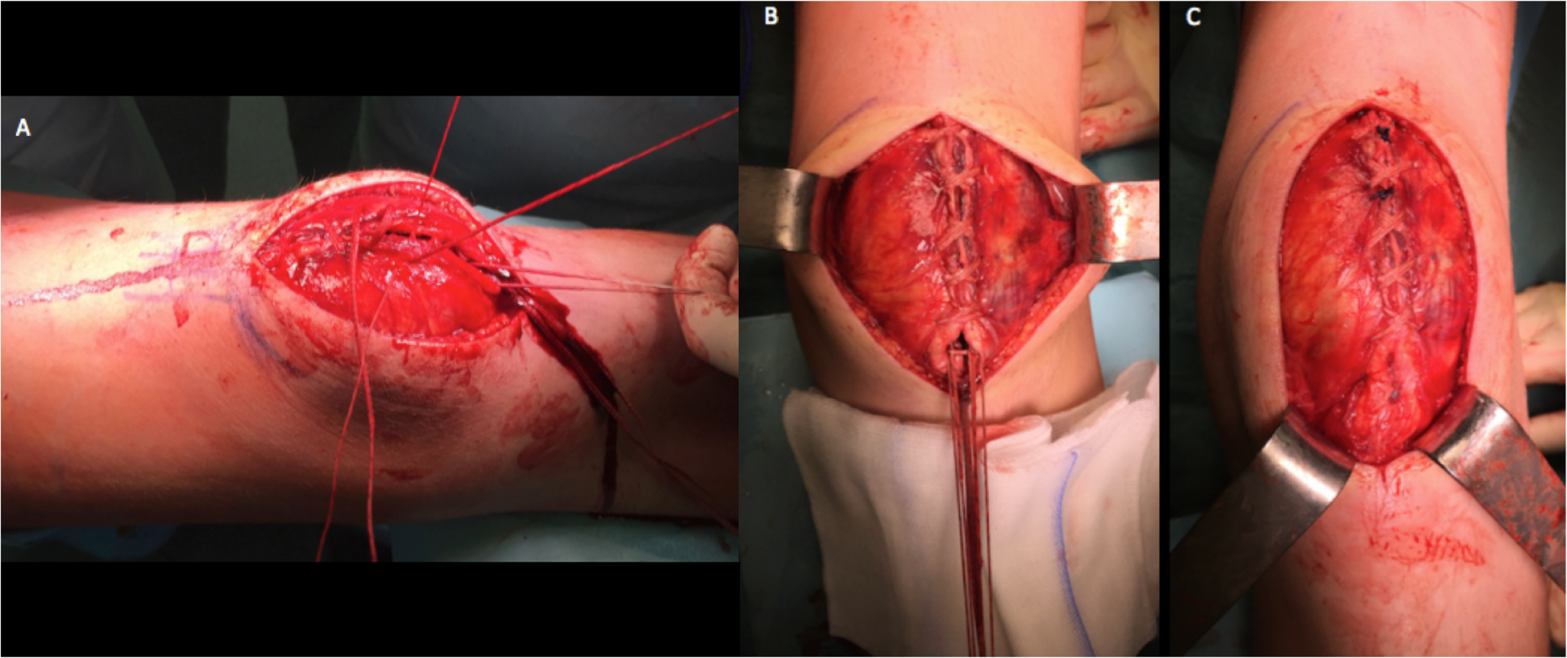
**a** and **b**: Shoelace suture of both ends of TigerTape™ closing the distal quadriceps tendon and periosteum over the patella. **c**: Final aspect of left knee after the TigerTape™ was tied onto the proximal patellar tendon and hidden beneath the periosteum

Under general anesthesia, the PSA was fixed with double trans osseous ultra-high strength tapes. A midline skin incision was used. The subcutaneous layer and the bursa were incised sharply showing a blood-infiltrated periosteum, which was incised longitudinally. Careful surgical exposure is mandatory to avoid transient ischaemic changes or an avascular necrosis of the proximal pole, because the blood supply of the immature patella originates predominantly from the anterior surface of the distal pole [8]. The entire periosteum was torn from inferior patella pole and peeled off the anterior patellar surface. The superior patellar pole and quadriceps tendon insertion were intact (Figs. 4a and b). Two vertical bone tunnels were drilled in the patella (Fig. 5a). A FiberTape® was first used, with one end passed through the lateral tunnel from distal to proximal and whipstitched the distal quadriceps tendon (Fig. 5b). A TigerTape™ suture (Arthrex, Naples, Florida) was then passed from distal to proximal, one end in each tunnel (Fig. 5c). The FiberTape® was shuttled back down the medial patellar tunnel and whipstitched the proximal patellar tendon where it was tied down in 90° of flexion (Fig. 5c). Both ends of the TigerTape™ were used to close the periosteum from proximal to distal over the anterior patellar surface in a shoestring suture technique and tied down over the proximal patellar tendon (Fig. 6a and b).

**Fig. 7:**
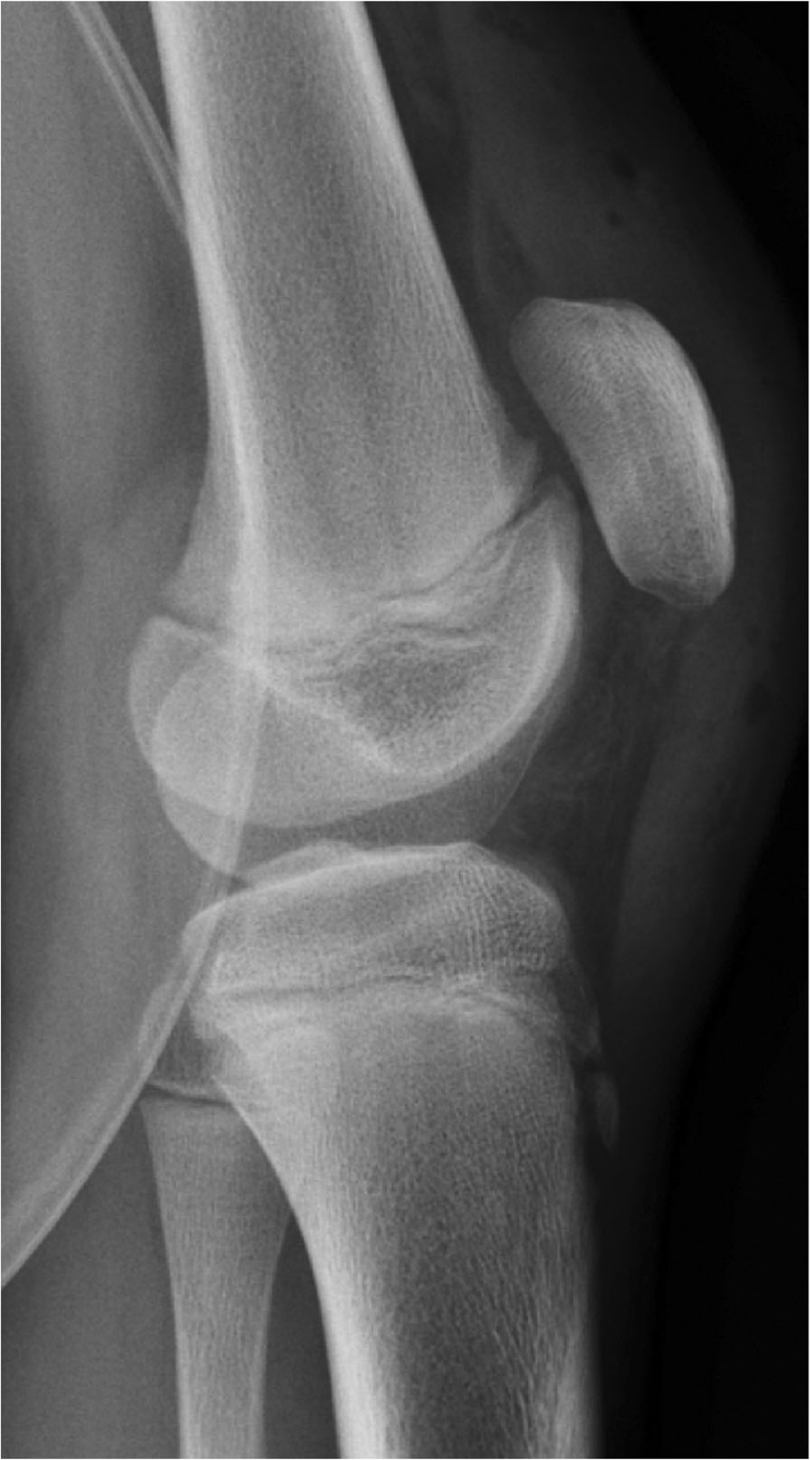
Postoperative X-ray shows a correct patellar height

**Fig. 8:**
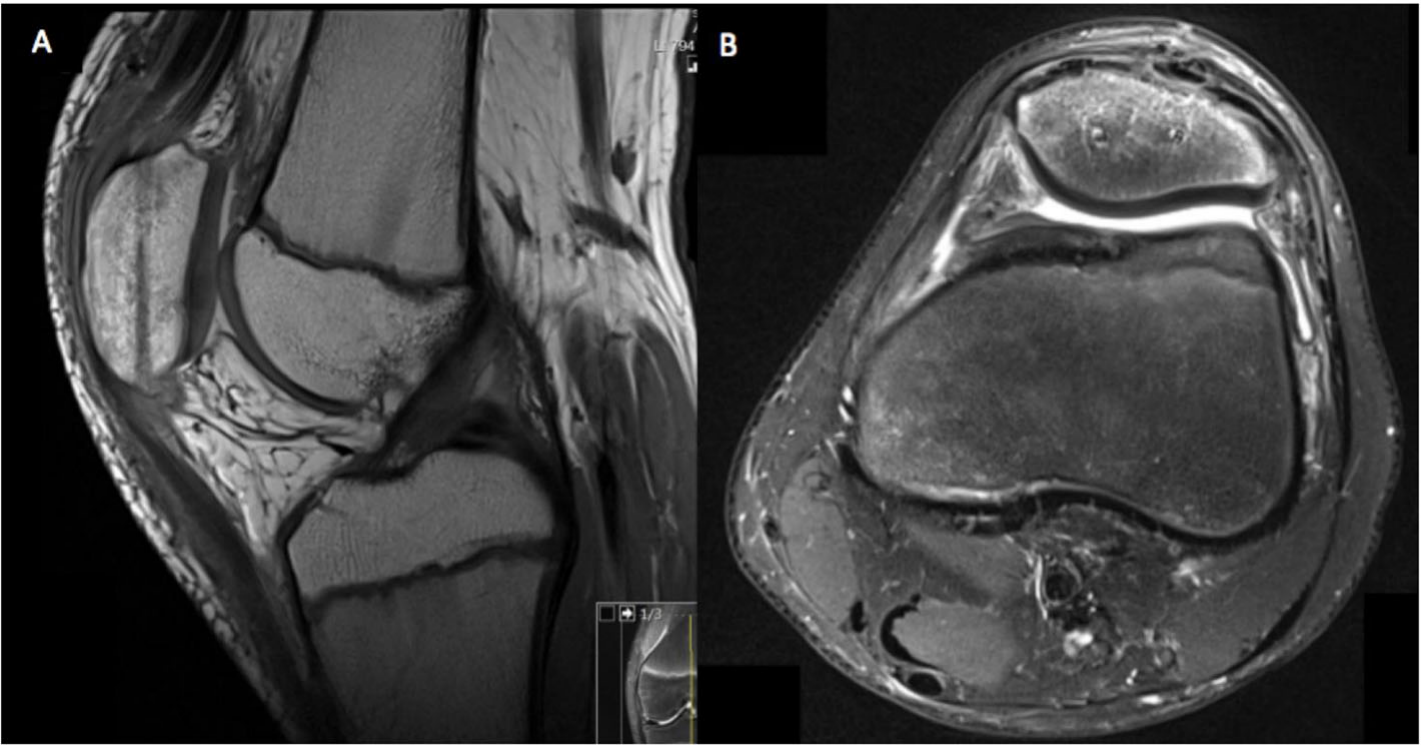
**a**, **b**: Postoperative T1-weighted sagittal and T2-weighted axial MR images showing healed periosteum and sleeve avulsion from inferior pole of the patella

Postoperatively, the knee was immobilized with a locked knee brace (M.4 X-lock®, MEDI) in full extension and weight bearing was restricted for 6 weeks. The postoperatively X-ray showed a correct patellar height (Fig. 7). Physical therapy was started immediately with full passive range of motion. At seven weeks postoperatively, the patient began active range of movement and partial weight bearing with 20 kg increments per week. At the 3-month follow- up visit the patient was able to ambulate brace free. Control MRI shows a healed periosteum and patellar tendon (Fig. 8). At 2-years follow up the patient described to be able to play soccer 3 times a week in the soccer club without pain. Ice hockey was also played without problems. Only slight pain by prolonged sitting and slight atrophy was described. The Kujala score at 2-years follow up was 96, Lysholm score 100, Tegner score 9, and VAS score 0.

## Discussion

The most important finding of the present case report is the occurrence of a PSA from the distal pole in a 12-year-old athlete with previous SLJ syndrome. Before acute PSA, our patient reported several months of anterior knee pain and tenderness over the inferior patellar pole after repetitive jumping activity.

In young patients, tibial tuberosity fractures are often preceded by various degrees of Osgood-Schlatter disease (OSD) [10]. Both OSD and SLJ syndrome are disorders seen in repetitive stress conditions, but there are no reports of SLJ syndrome with an increased fracture risk of the lower patellar pole. SLJ is an enchondral ossification disorder of the inferior pole of the patella, and is part of the osteochondrosis spectrum of diseases first described Sinding-Larsen and Johansson almost simultaneously in the 1920s [11]. Contrary to PSA, SLJ causes chronic pain and functional impairment of the knee in adolescent athletes in the same age group but without having suffered an acute trauma. Local inflammatory signs of the inferior pole of the patella and patellar tendon are present on physical examination [12]. Radiographic images of bone fragmentation in the distal pole appear within weeks of onset of symptoms, which have been described in 4 stages [13].

Sagittal MR images are useful in differentiating SLJ syndrome from PSA. Only a bony avulsion at the inferior patellar insertion is identified in the former entity, while the latter may present an extensive cartilaginous injury in addition to the osseous deformity [14] . The patient in this case had a complete disruption of the periosteum over the body of the patella with intact cartilage and a subtle bony avulsion in the inferior patellar pole and lateral retinaculum, barely visible on plain radiographs (Figs. 2, 3 and 4). Distinguishing between these entities is challenging and important with regard to patient treatment. Minimally displaced fractures such as those seen with SLJ syndrome are almost always managed conservatively and nonoperatively [15].

Closer attention to PSA fractures has been given only recently in literature since it was described in a case series by Houghton et al. in 1979 [8]. Only very rarely reported in adults [16], this entity is seen in skeletally immature patients that suffer an indirect pull of the extensor mechanism, where a small subchondral osseous fragment avulses together with an extensive sleeve of cartilage and periosteum from the main body of the patella. These fractures may occur in the entire circumference of the patella, and are described as superior, inferior, medial and lateral avulsions [9]. The unusual nature of our case is that the repetitive jumping mechanism led to a chronic overload of the extensor mechanism and ultimately to a PSA. It is not known if this entity is an advanced form of SLJ syndrome, and literature lacks studies that report direct causality.

Misdiagnosis is an unfortunate and frequent problem encountered with PSA. Prompt clinical diagnosis is important because clinical signs may be subtle. The clinicians should be aware of the risk factors and interrogate the patient thoroughly about the mechanism of injury. Not all patients have large effusions, decreased flexion or a clear disruption of the extensor mechanism. Non-displaced PSA with an intact posterior cartilaginous hinge will not necessarily lead to an active extensor lag. A high-riding patella or a palpable gap are clear signs of a displaced PSA in patients with severely swollen and tender knees [3]. Delay of diagnosis can result in suboptimal management and outcomes in displaced PSA [17]. If left untreated, a distal pull of the potent bone-forming tissue at the distal pole may go on forming bone and lead to an enlarged or even duplicated patella, active extensor lag and quadriceps muscle atrophy [3, 18].

Most authors agree that > 2 mm displaced PSA should be managed with open reduction and internal fixation in order to avoid the before mentioned complications. Treatment options vary because these fractures are uncommon, but good functional results are achieved with different techniques described in literature [2, 8, 9, 19]. The use of trans osseous nonabsorbable sutures alone are the preferred surgical treatment in our institution, in order to avoid a second step procedure seen in patients treated with cerclage wire augmentation [3] or tension band wiring with metal [20, 21]. We used two suture tapes (FiberTape® and TigerTape™) shuttled through 2 vertical bone tunnels in the patella in order to repair the avulsed periosteum and patellar tendon. We believe this technique is strong enough to withhold shear stress and eccentric loads allowing the patient to initiate an early rehabilitation regime. The use of non absorbable ultra strength sutures also decreases the risk of suture rejections and persistent wound drainage related to absorbable suture materials [8]. Absorbable and metal suture anchors [22, 23] and absorbable suture materials [24] are also frequently used surgical methods with good clinical outcomes.

## Conclusion

This paper and literature review has highlighted a case of distal pole PSA fractures in a young athlete with previous SLJ syndrome. Whether this syndrome seen in repetitive stress conditions increases the risk of sustaining a PSA is still not clear. Patella sleeve fractures in skeletally immature patients are difficult to detect and easy to overlook on plane radiographs. Awareness of typical characteristics of patient’s history, clinical examination and MRI are necessary for correct diagnosis. Early diagnosis and treatment with trans osseous fixation with suture tapes renders good functional results in a young athlete.

## Data Availability

All data concerning the case report are contained within the manuscript.

## Abbreviations

PSA: Patellar sleeve avulsion
MRI: Magnetic Resonance Imaging
SLJ: Sinding-Larsen-Johansson
X-ray: Plain radiographs
OSD: Osgood-Schlatter disease
